# Outcomes of Severe Acute Respiratory Diseases Caused by SARS-CoV-2, Influenza Virus, and Respiratory Syncytial Virus

**DOI:** 10.3390/microorganisms14071515

**Published:** 2026-07-11

**Authors:** Theofani Rimpa, Vasileios Skouras, Stefania Loli, Zacharias Diakonikolaou, Konstantina Dede, Konstantinos Eleftheriou, Apostolos Pappas, Ioannis Kalomenidis

**Affiliations:** 1 1st Department of Critical Care and Pulmonary Medicine, National and Kapodistrian University of Athens, Evaggelismos Hospital, Ιpsilantou 45-47, 10676 Athens, Greece; stefi1011@hotmail.com (S.L.); zadiak399@yahoo.gr (Z.D.); konsdede@gmail.com (K.D.); coselef@hotmail.com (K.E.); apo.pappas88@gmail.com (A.P.); ikalom@med.uoa.gr (I.K.); 2 Department of Pulmonary Medicine, 401 General Army Hospital, 11525 Athens, Greece; vskouras@otenet.gr

**Keywords:** RSV, COVID-19, Influenza, viral disease

## Abstract

COVID-19, influenza, and respiratory syncytial virus are leading causes of severe acute respiratory infection (SARI). As viral dynamics shift post-pandemic, comparing their clinical profiles is essential for optimizing management and vaccination strategies. We conducted a retrospective observational study of adults hospitalized with PCR-confirmed COVID-19, influenza, or RSV over three consecutive seasons (2021–2024). We compared clinical features, comorbidities and outcomes, including mortality, intubation rates and respiratory deterioration. Multivariable analyses were performed where virus was the independent variable and respiratory deterioration, intubation rate and mortality, Charlson Comorbidity Index, smoking status and pneumonia were co-variates. 263 patients were analyzed. Key findings revealed distinct clinical profiles: influenza patients were significantly younger, while COVID-19 was the predominant cause of viral pneumonia. RSV patients presented with the most severe oxygenation deficit upon admission and experienced the highest rate of respiratory deterioration (28%). Logistic regression analyses demonstrated that pneumonia [OR 2.81, 95% Confidence Interval (CI): 1.23–6.43, *p* = 0.01] and RSV (OR 2.8, 95% CI: 1.16–6.63, *p* = 0.02) were independently associated with respiratory deterioration. Similarly, pneumonia (OR 4.86, 95%CI: 1.62–17.36, *p* < 0.01] and RSV (OR 4.67, 95%CI: 1.5–14.34, *p* < 0.01) were independently associated with high risk of intubation. Among patients hospitalized because of COVID-19, RSV and Influenza, pneumonia and RSV were linked with respiratory deterioration and increased risk of intubation but not with death.

## 1. Introduction

Respiratory viral infections, including COVID-19 (SARS-CoV-2 virus), influenza, and infection with respiratory syncytial virus (RSV), are major causes of hospitalization in adults, especially older individuals and those with chronic diseases [1]. COVID-19 emerged in 2019 and caused a pandemic, resulting in millions of hospitalizations and deaths across all age groups [2]. Influenza infection peaks annually in the autumn and winter months, resulting in increased hospitalizations and fatalities. During the first year of the COVID-19 pandemic, the risk of death among hospitalized COVID-19 patients was substantially higher than that of patients hospitalized for seasonal influenza [3]. However, as the pandemic progressed, the risk of COVID-19–related mortality declined, and data from the most recent influenza season suggest that the numbers of hospitalizations and deaths due to influenza may have exceeded those of COVID-19 [4].

RSV has long been recognized as a common cause of respiratory infections. While it often causes mild, flu-like symptoms, RSV can also cause severe and even fatal respiratory disease [5]. Studies estimate that RSV infection accounts for 5–7% of respiratory infections among older or high-risk adult populations, with notable hospitalization and case-fatality rates [6]. Currently, there are no specific treatment options for RSV infection among adults, although vaccines are becoming available and therapeutic choices are under development [7].

While COVID-19, influenza, and RSV disease may present with similar symptoms and all three can cause fatal respiratory disease, information on whether they differ in terms of outcomes for patients with severe acute respiratory infection (SARI) [8] is still limited. In this study, we aimed to compare the clinical features and outcomes of patients hospitalized in a single center because of severe respiratory infection with any of the three viruses: influenza, COVID-19, and RSV. We hypothesized that clinical features, mortality, intubation rate, and duration of hospital stay would not differ between patients with different viral diseases.

## 2. Materials and Methods

This is a retrospective, observational study on patients hospitalized for COVID-19, RSV, or influenza conducted in our department during three consecutive influenza seasons (October–March, 2021–2024). The inclusion criteria were as follows: (1) age > 18 years old; (2) confirmed infection with any of the three viruses under investigation using pathogen-specific real-time reverse transcriptase polymerase chain reaction (RT-PCR) assays—Cepheid Xpert^®^ Xpress SARS-CoV-2/Flu/RSV assay (Cepheid, Sunnyvale, CA, USA)—performed on nasal swab specimens at the time of admission; (3) hospital admission because of respiratory manifestations of the infection (pneumonia or acute exacerbation of a chronic respiratory disease). The exclusion criteria were hospitalization for diseases unrelated to the viral infection and concomitant infection by more than one virus.

Eligible patients were identified from the digital hospital database with admission diagnoses. Patient data, including demographic characteristics, comorbidities, laboratory and imaging findings on admission, and outcomes, were extracted from their medical records. All patients received standard treatment according to international and national guidelines. The primary outcome was hospital mortality. Secondary outcomes included progression of respiratory failure (defined as the need for treatment with higher oxygen concentration or application of high-flow nasal oxygen or non-invasive ventilation), intubation, and duration of hospitalization. This study was approved by the Ethics Committee of Evaggelismos Hospital (Protocol no 71/10-3-2023). Given the retrospective design of this study, informed consent was not required.

### Statistical Analyses

Normally distributed continuous variables are presented as mean +/− standard deviation (SD) and compared using one-way ANOVA with Tukey’s post hoc test. Not normally distributed continuous variables are presented as medians (interquartile range, IQR) and compared using the Kruskal–Wallis test corrected with Dunn’s multiple-comparison test. Categorical variables are presented as numbers (percentages) and compared using the chi-square test or Fisher’s exact test as appropriate. The GraphPad Prism 10.0.0 software was used to perform statistical analysis. To investigate potential associations between different viral infections and certain outcomes, we created three different models, where viral infection was the independent variable, and respiratory deterioration, intubation rate, and mortality were the dependent variables in each model. Age, sex, CCI (Charlson Comorbidity Index), smoking status, and diagnosis of pneumonia at admission were included as covariates. A G-squared log-likelihood test was used to evaluate the goodness of fit. Missing data were handled using a listwise case deletion approach. Collinearity was estimated using the variance inflation factor. Values greater than 2 were considered indicators of collinearity. For every logistic regression analysis, the degrees of freedom for the intercept model (*n* − 1) and for the selected model (*n* − *k* − 1) were calculated. N represents the number of participants, and k represents the number of selected variables. The significance level (*p*-value) for the study was set to 0.05, and all tests were two-sided.

## 3. Results

### 3.1. Patients’ Clinical Features on Admission

A total of 263 patients were included in the study. Among them, 137 were hospitalized due to respiratory complications of COVID-19 infection, 87 due to influenza, and 39 due to RSV infection. Of the 87 patients hospitalized for complications due to influenza, 77 had influenza type A, and the rest had type B. In Table 1, we present the clinical characteristics of patients at the time of admission. Due to missing data, analyses of smoking status, body mass index (BMI), comorbidities (Charlson Comorbidity Index—CCI), and duration of symptoms were conducted for 241, 258, 253, and 238 patients, respectively. Patients with influenza were significantly younger and more likely to be smokers compared to those in the other viral groups. COVID-19 patients presented mainly with pneumonia, while patients in the two other groups presented with acute exacerbation of asthma or COPD. COVID-19 patients were significantly more likely to present with pneumonia compared to RSV patients but significantly less likely to present with acute exacerbation of COPD compared to influenza patients. COVID-19 patients had a significantly higher risk of exhibiting bilateral radiographic findings compared to influenza and RSV patients.

COVID-19 vaccination rates declined over time, from 31.9% in 2022–2023 to 2.4% in 2024–2025 (Supplementary Table S1). None of the patients had been vaccinated for RSV, and information about flu vaccination was not found.

In the subgroup of patients with pneumonia (Supplementary Table S2), influenza patients were significantly younger than RSV patients, more COVID-19 and RSV patients were males compared to influenza patients, and the CCI of patients with influenza was lower than the CCI of RSV patients. In the subgroup of patients with COPD exacerbation, the age of influenza patients was younger compared to RSV patients, and their CCI was significantly higher compared to the COVID-19 and RSV group (Supplementary Table S3). In the subgroup of patients with asthma exacerbation, no significant differences were found in terms of clinical features between groups (Supplementary Table S4).

### 3.2. Laboratory Values

The laboratory markers on admission that were statistically significant among the three viral groups were as follows: the PaO_2_/FiO_2_ ratio on admission of RSV patients was significantly lower compared to patients with influenza; the number of platelets in patients with influenza was lower compared to the other two groups; the value of SGOT in COVID-19 patients was significantly lower than in influenza patients; fibrinogen in COVID-19 patients was higher compared to patients with influenza; the urea levels in patients with influenza were lower than in COVID-19 patients; the CRP levels in RSV patients were lower compared to those in COVID-19 patients (Table 2).

Regarding the laboratory results in the subgroup of patients with pneumonia, the white blood cell counts in COVID-19 patients were significantly lower compared to RSV patients, and the urea levels in COVID-19 patients were significantly higher compared to RSV patients (Supplementary Table S5). In the COPD subgroup, RSV patients had significantly lower PaO_2_/FiO_2_ compared to COVID-19 and influenza patients; the SGOT levels in COVID-19 patients were lower compared to influenza patients; bilirubin levels in RSV patients were higher than in COVID-19 patients; and fibrinogen levels in influenza patients were significantly lower than in COVID-19 and RSV patients (Supplementary Table S6). However, in the asthma subgroup, the CRP levels in patients with asthma exacerbation due to influenza were significantly higher compared to the COVID-19 and RSV groups (Supplementary Table S7). Documented secondary bacterial superinfections occurred in 5.3% of the patients during hospitalization. The majority of these superinfections were observed in patients in the COVID-19 group (85.7%), whereas the remaining cases were evenly distributed between the influenza and RSV groups (Table 2).

### 3.3. Outcomes

During their hospitalization, a higher proportion of RSV patients presented respiratory deterioration compared to influenza patients. While the intubation rate tended to be higher in RSV patients compared to the other groups and mortality tended to be higher in COVID-19 and RSV compared to influenza, these differences were not statistically significant (Table 3).

Based on the initial findings identifying RSV infection as a potential risk factor for worse clinical outcomes, we developed multiple logistic regression models to investigate whether RSV infection was linked to a higher risk of respiratory deterioration, intubation, or mortality. We observed that RSV infection and a diagnosis of pneumonia at admission were independently associated with an increased risk of respiratory deterioration (Table 4) and a higher intubation rate (Table 5). Regarding a potential link between RSV infection and mortality, we found that only 1/94 of non-smokers died, which prevented us from fitting a logistic regression model with the preselected covariates because of perfect separation. For this reason, we performed the analysis again by removing smoking status from the final model. Although we observed that RSV infection increased the risk for death, this finding did not reach statistical significance in the final model (Table 6).

In the subgroup of patients with pneumonia, a significantly higher proportion of RSV and COVID-19 patients experienced respiratory deterioration during their hospitalization compared to influenza patients (Supplementary Table S8). Patients with pneumonia due to RSV presented higher, though not significantly different, intubation and mortality rates compared to COVID-19 and influenza patients (Supplementary Table S8). The outcomes of patients with COPD or asthma exacerbation (respiratory deterioration, length of hospitalization, intubation, and mortality) were similar across the three viral groups (Supplementary Tables S9 and S10). However, patients with acute COPD exacerbation due to RSV presented a higher rate of respiratory deterioration, intubation, and mortality, even though the difference was not significant (Supplementary Table S9).

## 4. Discussion

The present study aimed to identify and compare the characteristics and outcomes of patients hospitalized with respiratory complications of COVID-19, RSV disease, and influenza. Our main findings were as follows: (i) influenza patients were younger than patients with COVID-19 or RSV; (ii) RSV patients were predominantly of the female gender, while COVID-19 and influenza patients were predominantly male; (iii) pneumonia was the most common presentation only in the COVID-19 group; (iv) RSV patients presented with more severe hypoxia on admission and were more likely to have respiratory deterioration compared to influenza patients, while these clinical features did not significantly differ between COVID-19 and RSV patients; (v) multiple logistic regression analyses showed that RSV disease and a diagnosis of pneumonia at admission were independently associated with an increased risk of respiratory deterioration and a higher intubation rate but not an increased risk for hospital death.

No difference was observed regarding the primary outcome of this study; mortality was similar across the three viral groups, either when examined as a whole sample or when patients were evaluated according to different virus-induced respiratory complications (pneumonia, asthma, or COPD exacerbation). A recent cohort study across two respiratory seasons showed no difference in 30-day mortality during the 2023–2024 season, while the 30-day mortality rate of COVID-19 patients was higher compared to that of influenza patients during the 2022–2023 season [1]. A large cohort study of US veterans that tested all three viral groups concurrently found that COVID-19 was associated with higher mortality [9], and another study that compared older adults hospitalized with acute respiratory symptoms found that 30-day mortality was highest in RSV, followed by COVID-19, and lowest in influenza [10]. As for the intubation rate, although we did not observe statistically significant differences between the groups, it was high in RSV patients (18%), nearly double that of COVID-19 (10%), and more than double that of influenza (7%). Recent multicenter US studies show that the proportion of hospitalized patients requiring invasive mechanical ventilation is approximately 14.1% for COVID-19, 12.0% for RSV, and 10.3% for influenza [1]. Another study suggests that in older adults, RSV is less common but associated with more severe disease and a higher intubation rate than influenza [1,10,11]. The observed differences in intubation rate and mortality between studies may be explained by different patients’ characteristics, including age, comorbidities, socioeconomic status, access to health care, vaccination rates, or differences in available hospital resources between different periods and countries.

As for the secondary outcomes, RSV disease and a diagnosis of pneumonia on admission were independently associated with an increased risk of respiratory deterioration and a higher intubation rate, but not an increased risk for hospital death. Our findings are in agreement with the results of two recent studies, which showed that RSV patients were more likely to receive supplemental oxygen therapy or HFNC during their hospitalization compared to COVID-19 and influenza patients [9,11]. These studies also showed that in hospitalized adults aged ≥60 years, RSV was associated with more severe disease than both COVID-19 and influenza, with 12.0% experiencing intubation or death [9,11]. Older studies using data from 2020 to 2022 have demonstrated that patients hospitalized with RSV were less likely to experience mechanical ventilation and death than were patients hospitalized with COVID-19 [12,13]. This disparity should most likely be attributed to the increased population, anti-SARS-CoV2 immunity from both vaccination and infection, and improvements in the standard of care for COVID-19. More interestingly, in the subgroup of patients with pneumonia, both RSV and COVID-19 patients were significantly more likely to experience respiratory deterioration (47% and 27%, respectively) compared to those with pneumonia due to influenza (8%). It is reasonable to assume that bilateral involvement could be on the causal pathophysiologic path to worse oxygenation and deterioration, since it suggests reduced baseline lung reserve (on admission). It is not surprising, therefore, that a higher proportion of extensive parenchymal involvement (bilateral infiltrates) in RSV and COVID-19 coincides with the most common respiratory deterioration observed in the RSV group (47%) and in COVID-19 (27%) compared to that in influenza (5%). The severity of respiratory disease due to RSV highlights the importance of RSV vaccination and treatment. RSV vaccines are highly effective in preventing lower respiratory tract disease in adults aged 60 and older [14,15]. In contrast to advances in RSV vaccination, treatment remains limited to supportive care. While monoclonal antibodies such as nirsevimab and clesrovimab show promise, they are currently approved only as a preventive measure in infants [16,17]. Ongoing trials investigating antivirals—including ribavirin, molnupiravir, and favipiravir—may soon provide the evidence required to address the current limitations in adult RSV treatment [18,19,20].

Analysis of the demographic characteristics and clinical presentation of the patients also unveils some interesting differences between the three groups. First, a significant age gap was observed between the three viral groups: influenza patients were nearly a decade younger than those in the COVID-19 and RSV groups, while RSV mainly affected older and mostly female patients. Other studies demonstrated that COVID-19 affects all age groups, but hospitalization rates are higher in adults aged ≥65 years and infants under 1 year [21,22], while RSV predominantly affects infants and young children—especially those under 6 months of age—who have the highest rates of hospitalization [20,23], and influenza hospitalization rates are elevated in both young children and older adults [1,10]. Second, in this study, the influenza group presented a substantially higher prevalence of smoking (64%). Smoking has been previously shown to be a risk factor for acquiring influenza, increased disease severity, and hospitalization [24,25,26,27]. Concerning the COVID-19 vaccination status, a marked decline in the proportion of documented vaccinated patients was noted during the study period (Supplementary Table S1). This drop in vaccinated admissions correlates with the progressive displacement of older Omicron subvariants by the JN.1 lineage (winter 2023–2024) and subsequent KP.3.1.1/XEC strains (2024–2025) [28]. The Charlson Comorbidity Index (CCI) was lower in the COPD subgroup with influenza compared to the other viruses, which highlights the impact of influenza on the hospitalization risk of COPD patients even with few or no comorbidities. This observation is in line with the findings of a population-based cohort study that showed that influenza infection carries an increased risk of acute exacerbation in COPD patients, independent of the presence of other comorbidities [29,30].

In our study, COVID-19 was the leading cause of pneumonia, accounting for two out of three (93 out of 147) cases of viral pneumonia. This finding agrees with other studies demonstrating that COVID-19 remains the leading cause of viral pneumonia in adult hospitalized patients, even after the pandemic [1,31]. In contrast, RSV pneumonia is more common in the pediatric population and older adults, especially those with comorbidities, while influenza remains a major cause of pneumonia in both adults and children [1,11,31,32]. In this study, COPD exacerbations were less common in COVID-19 patients (24%) compared to influenza (45%) and RSV (41%) patients, while the rate of asthma exacerbations was higher in RSV patients (20%) compared to COVID-19 (8%) and influenza (10%) patients, although the difference was not statistically significant. Another study suggests that RSV infection is associated with higher COPD exacerbation rates compared to influenza; among hospitalized older adults, RSV infection carries a 1.7-fold increased odds of COPD exacerbation compared to influenza [33]. Another study on Danish adults found that RSV infection is associated with a 3.1-fold increased relative risk of COPD exacerbations compared to matched unexposed individuals [34]. Concerning asthma exacerbations, in a Danish nationwide study, RSV infection carried a 4.6-fold increased relative risk of asthma exacerbations compared to matched unexposed individuals, while influenza showed a lower relative risk [34]. In contrast, COVID-19 is not commonly implicated as a cause of acute exacerbation of a chronic obstructive disease [35]. COVID-19-related COPD exacerbations are also associated with higher rates of ICU admission, need for respiratory support, longer hospital stays, and increased mortality compared to other viruses [35].

Several limitations of the present study must be acknowledged. The most important limitation is its retrospective nature, which resulted in missing values. However, there were no missing values regarding the clinical outcomes of the patients, which were the endpoints of this study. In addition, the low number of RSV patients may have limited the statistical power of this study to unveil significant differences in clinical outcomes, such as intubation and mortality rates between RSV and (mainly) influenza patients, especially when subgroups with different respiratory abnormalities were assessed. To obtain an adequate sample size and mitigate potential bias related to seasonal fluctuations in clinical workload, since RSV and influenza but not COVID-19 present substantial seasonality, our study period included three consecutive influenza (and RSV) seasons instead of whole years. Furthermore, this study did not take into consideration possible differences in the virus strains that were in circulation or the vaccination status of the patients. From a methodological viewpoint, comparing multiple groups in univariate analyses presents a risk of false-positive findings, especially in regard to parameters showing statistically significant differences, i.e., respiratory deterioration. However, it should be noted that the significance of the link between RSV infection and outcomes was assessed with multivariable logistic regression analysis, which is a more powerful statistical method than the chi-square and Fisher’s exact tests.

## 5. Conclusions

In conclusion, the clinical presentation and progression of the three viruses present distinct profiles. RSV disease was found to be linked to higher rates of respiratory deterioration and intubation, while the risk of hospital death was similar in the three groups of patients with viral respiratory diseases. Our findings highlight the necessity for the development of new therapeutic agents targeting RSV.

## Figures and Tables

**Table 1:** Demographic characteristics and baseline features of patients hospitalized with COVID-19, RSV and influenza.

	COVID-19 (Ν = 137)	RSV (Ν = 39)	Influenza (Ν = 87)	*p*-Value
Age, years	73 (65–80)	75 (65–79)	66 (58–74) *#	<0.0001
Male gender	91 (66%)	15 (38%) *⇞	44 (51%)	0.0026
Smoking status				
smoker	36 (26%)	15 (38%)	56 (64%) *#	0.007
ex-smoker	53 (39%)	17 (44%)	16 (18%)	NS
non-smoker	34 (25%)	7 (18%)	7 (8%)	NS
Body Mass Index > 30 kg/m^2^	12 (9%)	6 (15%)	5 (6%)	NS
Charlson Comorbidity Index	3 (2–5)	3 (2–4)	4 (2–5)	NS
Pneumonia	93 (68%)	15 (38%) *	39 (45%)	<0.0001
Bilateral pneumonia	44 (32%) ⇞	5 (13%)	10 (11%)	<0.0001
Acute Exacerbation of COPD	33 (24%)	16 (41%)	39 (45%) *	0.004
Acute Exacerbation of Asthma	11 (8%)	8 (20%)	9 (10%)	NS
Duration of symptoms	3 (2–7)	4 (2–5)	3 (1–5)	NS

Quantitative data are presented as median (interquartile range) and qualitative data as number (%); RSV: Respiratory Syncytial Virus; COPD: Chronic Obstructive Lung Disease; *: *p* < 0.05 vs. COVID-19; #: *p* < 0.05 vs. RSV; ⇞: *p* < 0.05 vs. influenza; NS: Not Statistically Significant.

**Table 2:** Laboratory values and respiratory parameters on admission of patients with COVID-19, RSV and influenza.

	COVID-19 (Ν = 137)	RSV (Ν = 39)	Influenza (Ν = 87)	*p*-Value
White blood cells, /μL	9010 (6915–12,335)	9350 (7480–12,710)	9700 (6610–12,570)	NS
Lymphocytes, /μL	1110 (740–1655)	1290 (810–1480)	1090 (660–1570)	NS
Platelets, ×1000/μL	238 (180–298)	250 (190–306)	214 (166–261)	0.034
Hemoglobulin, g/dL	13 (11.35–14.10)	13 (11.50–15.00)	13.30 (12.40–14.30)	NS
SGOT, UL/L	26 (19–38) ⇞	27 (20–42)	29 (22–46)	0.049
SGPT, UL/L	19 (12.50–30.50)	20 (17–34)	22 (16–35)	NS
Bilirubin, mg/dL	0.46 (0.46–0.73)	0.61 (0.39–0.88)	0.46 (0.33–0.69)	NS
D-dimers, mg/dL	0.95 (0.36–1.88)	0.67 (0.43–3.98)	0.95 (0.57–1.43)	NS
Fibrinogen, mg/dL	603 (496–772) ⇞	560 (461–677)	509 (435–627)	<0.001
Urea, mg/dL	45 (33–66)	39 (28–49)	34 (28–47) *	0.001
Creatinine, mg/dL	0.90 (0.70–1.30)	0.80 (0.70–1.00)	0.80 (0.70–1.10)	NS
CRP, mg/dL	9.10 (2.80–22.90)	4.30 (1.40–10.50) *	6.60 (3.40–17.60)	0.021
PaO_2_/FiO_2_	261 (238–300)	241.5 (185–281.5) ⇞	271 (238–308.5)	0.047
Bacterial co-infection	12 (8.8%) ⇞	1 (2.6%)	1 (1.1%)	0.03

Data are presented as median (interquartile range); RSV: Respiratory Syncytial Virus; SGOT: Serum Glutamic-Oxaloacetic Transaminase; SGPT: Serum Glutamic Pyruvic Transaminase; CRP: C-Reactive Protein; * *p* < 0.05 vs. COVID-19; ⇞: *p* < 0.05 vs. influenza; NS: Not Statistically Significant.

**Table 3:** Clinical outcomes (respiratory deterioration, intubation, mortality) among three infection groups.

	COVID-19 (Ν = 137)	RSV (Ν = 39)	Influenza (Ν = 87)	*p*-Value
Respiratory deterioration	23% (0.164–0.303)	28% (0.165–0.438) ⇞	13% (0.055–0.185)	0.049
Length of hospitalization	8 (5.5–12.5)	7 (5–12)	8 (6–10)	NS
Intubation % (95% CI)	10% (0.619–0.164)	18% (0.092–0.334)	7% (0.032–0.142)	NS
Mortality	9% (0.051–0.147)	10% (0.041–0.236)	3% (0.012–0.097)	NS

Quantitative data are presented as median (interquartile range) and qualitative data as percentages (%) alongside their corresponding 95% confidence intervals in parentheses; RSV: Respiratory Syncytial Virus; ⇞: *p* < 0.05 vs. influenza; NS: Not Statistically Significant.

**Table 4:** RSV infection is independently associated with higher risk of respiratory deterioration.

Dependent Variable: Respiratory Deterioration	Adjusted OR	95% CI	*p*-Value
Independent Variables			
Intercept	0.01	<0.01–0.14	<0.001
Age (per year)	1.03	0.99–1.07	0.15
Sex (Male)	1.01	0.48–2.16	0.98
CCI > 2	0.58	0.18–1.96	0.37
Smoker (ex or current)	1.67	0.65–4.63	0.31
Pneumonia at admission	2.81	1.23–6.43	0.01
RSV infection	2.8	1.16–6.64	0.02

A multiple logistic regression model was used to investigate the link between RSV infection and respiratory deterioration. Age, sex, CCI, smoking status and admission diagnosis were treated as co-variates. Degrees of freedom for the intercept-only model = 236 and for the selected model = 230, AU-ROC (CI) = 0.68 (0.59–0.78), *p* < 0.001. OR: Odds ratio, CI: Confidence intervals, CCI: Charlson’s comorbidity index, RSV: Respiratory syncytial virus, AU-ROC: Area under the receiver-operator characteristic curve; NS: Not Statistically Significant.

**Table 5:** RSV infection is independently associated with higher risk for intubation.

Dependent Variable: Intubation Rate	Adjusted OR	95% CI	*p*-Value
Independent Variables			
Intercept	0.03	<0.01–0.49	0.02
Age (per year)	0.97	0.93–1.02	0.21
Sex (Male)	1.1	0.39–3.25	0.86
CCI > 2	4.41	0.77–38.05	0.12
Smoker (ex or current)	2.28	0.59–11.67	0.27
Pneumonia at admission	4.86	1.62–17.36	<0.01
RSV infection	4.67	1.5–14.34	<0.01

A multiple logistic regression model was used to investigate the link between RSV infection and intubation rate. Age, sex, CCI, smoking status and admission diagnosis were treated as co-variates. Degrees of freedom for the intercept-only model = 236 and for the selected model = 230, AU-ROC (CI) = 0.75 (0.64–0.87), *p* < 0.001. OR: Odds ratio, CI: Confidence intervals, CCI: Charlson’s comorbidity index, RSV: Respiratory syncytial virus, AU-ROC: Area under the receiver-operator characteristic curve; NS: Not Statistically Significant.

**Table 6:** RSV infection is not independently associated with increased mortality.

Dependent Variable: Mortality	Adjusted OR	95% CI	*p*-Value
Independent Variables			
Intercept	<0.01	<0.01–0.02	<0.01
Age (per year)	1.06	0.99–1.15	0.12
Sex (Male)	0.97	0.3–3.25	0.96
CCI > 2	0.93	0.09–22.57	0.95
Pneumonia at admission	2.99	0.84–14.26	0.12
RSV infection	3.15	0.77–11.37	0.09

A multiple logistic regression model was used to investigate the link between RSV infection and mortality. Age, sex, CCI and admission diagnosis were treated as co-variates. Degrees of freedom for the intercept-only model = 251 and for the selected model = 246, AU-ROC (CI) = 0.75 (0.59–0.9), *p* < 0.001. OR: Odds ratio, CI: Confidence intervals, CCI: Charlson’s comorbidity index, RSV: Respiratory syncytial virus, AU-ROC: Area under the receiver-operator characteristic curve; NS: Not Statistically Significant.

## Data Availability

The original contributions presented in this study are included in the article/Supplementary Materials. Further inquiries can be directed to the corresponding author.

## Supplementary Materials

The following supporting information can be downloaded at https://www.mdpi.com/article/10.3390/microorganisms14071515/s1. Table S1: COVID-19 Vaccination Status of Hospitalized Patients across Three Consecutive Periods; Table S2: Demographic characteristics and baseline features of patients hospitalized with pneumonia due to COVID-19, RSV and influenza; Table S3: Demographic characteristics and baseline features of patients hospitalized with COPD exacerbation due to COVID-19, RSV and influenza; Table S4: Demographic characteristics and baseline features of patients hospitalized with asthma exacerbation due to COVID-19, RSV and influenza; Table S5: Laboratory values and respiratory parameters on admission of patients with pneumonia due to COVID-19, RSV and influenza; Table S6: Laboratory values and respiratory parameters on admission of patients with COPD exacerbation due to COVID-19, RSV and influenza; Table S7: Laboratory values and respiratory parameters on admission of patients with asthma exacerbation due to COVID-19, RSV and influenza; Table S8: Outcomes among patients with pneumonia due to COVID-19, RSV or influenza; Table S9: Outcomes among patients with COPD exacerbation infected with COVID-19, RSV or influenza; Table S10: Outcomes among patients with asthma exacerbation infected with COVID-19, RSV or influenza.
