# Supplementary material for: Outcomes of Severe Acute Respiratory Diseases Caused by SARS-CoV-2, Influenza Virus, and Respiratory Syncytial Virus
Source: Microorganisms. 2026 Jul 11;14(7):1515. doi: 10.3390/microorganisms14071515 (PMC13414054; doi:10.3390/microorganisms14071515)
Supplement: Supplementary file 1 [file microorganisms-14-01515-s001.zip › microorganisms-4367213-supplementary.pdf]

SUPPLEMENTARY DATA

Supplementary Table S1. COVID-19 Vaccination Status of Hospitalized Patients across Three Consecutive Periods

Time Period	Documented Vaccinated	Confirmed Unvaccinated	Unknown / Undocumented
**2022–2023**	36 (31.9%)	32 (28.3%)	45 (39.8%)
**2023–2024**	3 (4.8%)	32 (50.8%)	28 (44.4%)
**2024–2025**	2 (2.4%)	26 (30.6%)	57 (67.1%)

Quantitative data are presented as median (interquartile range) and qualitative data as number (%)

Supplementary Table S2. Demographic characteristics and baseline features of patients hospitalized with pneumonia due to COVID-19, RSV and influenza

	COVID-19 (n=93)	RSV (n=15)	Influenza (n=39)	p-value
**Age, years**	74 (66-85)	76 (69-81)	65 (56-74)#	<0.001
**Male gender**	60 (65%)	6 (40%)*ǂ	18 (47%)	<0.001
**Body Mass Index>30**	6 (6%)	1 (6%)	1 (2%)	NS
**Charlson Comorbidity Index**	4 (3-5)	4 (4-5)	3 (2-4.5)	0.049
**Duration of symptoms**	4 (2-8)	4.5 (1.75-6.25)	4 (2-6.75)	NS

Quantitative data are presented as median (interquartile range) and qualitative data as number (%); RSV: Respiratory Syncytial Virus; *p<0.05 vs COVID-19; #p:<0.05 vs RSV; ǂp:<0.05 vs influenza

Supplementary Table S3. Demographic characteristics and baseline features of patients hospitalized with COPD exacerbation due to COVID-19, RSV and influenza

	COVID-19 (n=33)	RSV (n=16)	Influenza (n=39)	p-value
**Age, years**	75 (67-80)	76 (68-79)	68 (60-74)#	0.044
**Male gender**	25 (75%)	8 (50%)	25	NS
**Body Mass Index>30**	5 (15%)	4 (25%)	2 (5%)	NS
**Charlson Comorbidity Index**	5 (4-6)	5 (4-6)	4 (3-5)*#	0.014
**Duration of symptoms**	3 (2-5)	3 (2-5)	2 (1-5)	NS

Quantitative data are presented as median (interquartile range) and qualitative data as number (%); RSV: Respiratory Syncytial Virus; *p<0.05 vs COVID-19; #p:<0.05 vs RSV;

Supplementary Table S4. Demographic characteristics and baseline features of patients hospitalized with asthma exacerbation due to COVID-19, RSV and influenza

	COVID-19 (n=11)	RSV (n=8)	Influenza (n=9)	p-value
**Age, years**	62 (48-73)	56 (39-70)	55 (45-64)	NS
**Male gender**	4 (36%)	1 (12%)	1 (11%)	NS
**Body Mass Index>30**	1 (9%)	1 (12%)	2 (22%)	NS
**Charlson Comorbidity Index**	5 (4-6)	5 (4-6)	4 (3-5)	NS
**Duration of symptoms**	1 (1-4)	3 (1-7)	2.5 (1-3)	NS

Quantitative data are presented as median (interquartile range) and qualitative data as number (%); RSV: Respiratory Syncytial Virus.

Supplementary Table S5. Laboratory values and respiratory parameters on admission of patients with pneumonia due to COVID-19, RSV and influenza

	COVID-19 (n=93)	RSV (n=15)	Influenza (n=39)	p-value
**White blood cells, /μL**	9010# (6925-12930)	12490 (9080-16860)	10630 (7150-12500)	0.026
**Lymphocytes, /μL**	1000 (670-1530)	1170 (900-1440)	1140 (780-1580)	NS
**Platelets, x1000/μL**	230 (179-301)	228 (188-333)	216 (168-255)	NS
**Hemoglobulin, g/dL**	12.50 (11.35-13.90)	12.40 (10.90-14.70)	12.90 (12.30-14.00)	NS
**SGOT, UL/L**	29 (21-42)	37 (20-56)	30 (22-46)	NS
**SGPT UL/L**	23 (13-34)	21 (16-49)	22 (18-34)	NS
**Bilirubin, mg/dL**	0.52 (0.39-0.77)	0.61 (0.43-0.92)	0.50 (0.34-0.79)	NS
**D-dimers, mg/dL**	1.40 (0.48-1.95)	0.59 (0.43-5.08)	0.99 (0.68-2.54)	NS
**Fibrinogen, mg/dL**	658 (522-826)	573 (430-743)	611 (498-716)	NS
**Urea, mg/dL**	51 (34-71) ǂ	42 (36-72)	39 (30-47)	0.016
**Creatinine, mg/dL**	1.00 (0.70-1.40)	0.90 (0.80-1.20)	0.90 (0.70-1.10)	NS
**CRP, mg/dL**	17.00 (5.65-25.80)	10.50 (3.50-18.90)	16.20 (5.40-21.60)	NS
**PaO2/FiO2**	252 (214-285)	216 (156-281)	271 (223-309)	NS

Data are presented as median (interquartile range) RSV: Respiratory Syncytial Virus; SGOT: Serum Glutamic-Oxaloacetic Transaminase:; SGPT: Serum Glutamic Pyruvic Transaminase; CRP:C-Reactive Protein; p<0.05 vs COVID-19; #p:<0.05 vs RSV; ǂp:<0.05 vs influenza

Supplementary Table S6. Laboratory values and respiratory parameters on admission of patients with COPD exacerbation due to COVID-19, RSV and influenza

	COVID-19 (n=33)	RSV (n=16)	Influenza (n=39)	p-value
**White blood cells, /μL**	9030 (6725-11540)	9290 (7800-11820)	8210 (5560-10750)	NS
**Lymphocytes, /μL**	1300 (830-1675)	1325 (730-1470)	1060 (590-1530)	NS
**Platelets, x1000/μL**	238 (174-292)	251 (188-230)	208 (164-263)	NS
**Hemoglobulin, g/dL**	13.70 (11.40-14.50)	13.10 (11.05-15.05)	13.40 (13.00-14.80)	NS
**SGOT, UL/L**	21 (16-33) ǂ	28 (22-37)	29 (24-46)	0.009
**SGPT UL/L**	16 (12-26)	20 (16-33)	21 (14-33)	NS
**Bilirubin, mg/dL**	0.39 (0.25-0.60)	0.62 (0.35-0.92)*	0.41 (0.30-0.60)	0.035

**D-dimers, mg/dL**	0.40 (0.28-0.73)	0.88 (0.74-1.01)	0.76 (0.54-1.47)	NS
**Fibrinogen, mg/dL**	558 (434-644)	580 (526-733)	472 (407-509)***#**	>0.001
**Urea, mg/dL**	44 (34-53)	38 (26-49)	34 (27-52)	NS
**Creatinine, mg/dL**	0.9 (0.70-1.10)	0.8 (0.70-0.98)	0.80 (0.70-1.10)	NS
**CRP, mg/dL**	3.2 (1.4-7.8)	5.2 (1.6-8.3)	4.4 (2.6-9.8)	NS
**PaO2/FiO2**	261 (241-288)	209 (184-277) ‡	276 (242-309)	0.011

Data are presented as median (interquartile range) RSV: Respiratory Syncytial Virus; SGOT: Serum Glutamic-Oxaloacetic Transaminase; SGPT: Serum Glutamic Pyruvic Transaminase; CRP:C-Reactive Protein; *p<0.05 vs COVID-19; #p:<0.05 vs RSV; ‡p:<0.05 vs influenza

Supplementary Table S7. Laboratory values and respiratory parameters on admission of patients with asthma exacerbation due to COVID-19, RSV and influenza

	**COVID-19 (n=11)**	**RSV (n=8)**	**Influenza (n=9)**	**p-value**
**White blood cells, /μL**	8850 (7020-12010)	7230 (5578-8993)	12060 (7765-18230)	NS
**Lymphocytes, /μL**	1980 (1140-2630)	1410 (668-2075)	870 (495-1875)	NS
**Platelets, x1000/μL**	284 (239-320)	275 (211-299)	248 (160-298)	NS
**Hemoglobulin, g/dL**	13.20 (11.10-14.60)	13.80 (11.95-13.98)	13.40 (11.85-14.85)	NS
**SGOT, UL/L**	18 (14-24)	21 (17-26)	29 (21-53)	NS
**SGPT UL/L**	17 (8-19)	21 (18-30)	32 (13-44)	NS
**Bilirubin, mg/dL**	0.34 (0.25-0.42)	0.49 (0.25-0.88)	0.47 (0.35-0.78)	NS
**D-dimers, mg/dL**	0.49 (0.26-0.72)	0.41 (0.41-0.41)	0.58 (0.20-0.95)	NS
**Fibrinogen, mg/dL**	409 (379-495)	430 (324-557)	523 (378-583)	NS
**Urea, mg/dL**	34 (25-48)	30 (21-39)	28 (23-43)	NS
**Creatinine, mg/dL**	0.70 (0.65-0.80)	0.75 (0.63-0.95)	0.70 (0.0.65-0.80)	NS
**CRP, mg/dL**	1.00 (0.40-13.00)	1.35 (0.23-2.03)	6.60 (4.55-21.35)#	0.004
**PaO2/FiO2**	295 (221-337)	293 (191-373)	266 (238-333)	NS

Data are presented as median (interquartile range) RSV: Respiratory Syncytial Virus; SGOT: Serum Glutamic-Oxaloacetic Transaminase:; SGPT: Serum Glutamic Pyruvic Transaminase; CRP:C-Reactive Protein; * p<0.05 vs COVID-19; #p:<0.05 vs RSV

Supplementary Table S8. Outcomes among patients with pneumonia due to COVID-19, RSV or influenza

	**COVID-19 (n=93)**	**RSV (n=15)**	**Influenza (n=39)**	**p-value**
**Respiratory deterioration**	27% (0.199-0.378)‡	47% (0.248-0.699)‡	5% (0.014-0.169)	0.04
**Length of hospitalization**	9 (6-13)	7 (5-13)	8 (6-12)	NS
**Intubation**	13% (0.075-0.212)	33% (0.152-0.583)	13% (0.056-0.267)	NS
**Mortality**	11% (0.067-0.199)	20% (0.07-0.452)	5% (0.014-0.169)	NS

Quantitative data are presented as median (interquartile range) and qualitative data as percentages (%) alongside their corresponding 95% confidence intervals in parentheses; RSV: Respiratory Syncytial Virus; ‡p<0.05 vs influenza

Supplementary Table S9. Outcomes among patients with COPD exacerbation infected with COVID-19, RSV or influenza

	COVID-19 (n=33)	RSV (n=16)	Influenza (n=39)	p-value
**Respiratory deterioration**	12% (0.067-0.309)	19% (0.066-0.43)	13% (0.072-0.297)	NS
**Length of hospitalization**	9 (6-10)	9 (6-10)	9 (6-10)	NS
**Intubation**	3% (0.005-0.153)	13% (0.035-0.36)	3% (0.005-0.153)	NS
**Mortality**	3% (0.005-0.153)	6% (0.011-0.283)	3% (0.005-0.132)	NS

Quantitative data are presented as median (interquartile range) and qualitative data as percentages (%) alongside their corresponding 95% confidence intervals in parentheses; RSV: Respiratory Syncytial Virus

Supplementary Table S10. Outcomes among patients with asthma exacerbation infected with COVID-19, RSV or influenza

	COVID-19 (n=11)	RSV (n=8)	Influenza (n=9)	p-value
**Respiratory deterioration**	9% (0.016-0.377)	13% (0.022-0.471)	0% (0-0.299)	NS
**Length of hospitalization**	6 (5-10)	6 (4-11)	7 (6-9)	NS
**Intubation**	9% (0.016-0.377)	0% (0-0.324)	0% (0-0.299)	NS
**Mortality**	0% (0- 0.259)	0% (0-0.324)	0% (0-0.299)	NS

Quantitative data are presented as median (interquartile range) and qualitative data as percentages (%) alongside their corresponding 95% confidence intervals in parentheses; RSV: Respiratory Syncytial Virus
